# Supplementary material for: Repetitive Sequence Barcode Probe for Karyotype Analysis in Tripidium arundinaceum
Source: Int J Mol Sci. 2022 Jun 16;23(12):6726. doi: 10.3390/ijms23126726 (PMC9224303; doi:10.3390/ijms23126726)
Supplement: Supplementary file 1 [file ijms-23-06726-s001.zip › ijms-1759696-supplementary.pdf]

Table S1. Nucleotide sequence of probes.

>Ea-0098

AGTTTCCACGGTAGCACGAGGACGTGGTGATGGTGCTTGACCACACCTGCTTCACATGTTCA
TGTACCCATTGCACCGCAGAGGCACACCAACAATCCGGTTCAACATCAAAGAACAAAGCATA
TTGATGTTCGTCATCATTTCATTAGAGATCATCAACAAAAGGGTGACATTGCTATTGAGAGTGT
GGGCACCGAAGATCAA CTAGCCGATCTATTCACCAAGCCACTTGATGAGAAGAGATTTC

>Ea-0907

TGGAATCATTGTTATTGTTGGTGCAAGATCGGGGCATGGTTTGCGCCGAACGTGATAGCTTGAT
GTTCTTGTGCCTTTTTCCTGCTGTACTGTGACTTCGTTTGTGTCCAGGCTCGCGTCTCTAGCACG
GTCTAGCCTAGGACCAGTCAAATTATCGAGCTCCAGAAGACTGATGTGGGTGTGTTTCACATC
AAGCGGAAGATGAAAGAGCAAGAAACCAAGCACTTTCGAGTGGATGACAAAGGAATTCTC
AGGTTCAACGAGTGGTTCCCGGCCACACAGATGCCTGGATTCACTCCCGGGTATCAGCAGGG
CTGGGAACACACCCCGCAACCACACGAGGCCGGAGGATCGGGATGGCACGAGGCCATGTT
GAACCTTTTCACATTCCTCATTGGTGAGACTTTCAAGTAGACATAATCCCCTACTTCAAAAGTC
AACTCTCTCCTTCGAGTATCAGCATAACTTTTCTGCCGGGACTAAGCGATCTTTAGATTATCTCT
AAATCGAGTATTTTCTTCACCGGGGCTTGCCAAAGAGGCCGTTTTTGGGACTCAGTTTGATTT
CGCGCGAC

>Ea-0663

AGAATTCGGATCCAAGCTTCTGGTTTGTCTTCTTCATCAACTAGTTAACCATCAATTTGAACAACTA
GCCCAAAGGGAAAGTGAGTAAATAGTGCCCGAATCGAGTATTTTCACAAACCAGAAGCTTGGATC
CGAATTCT

>Ea-0070

TATCACGAGGTAGGTGGTGGAGGCGGCAGCGATGGGATTTGGGGTTAAAGTTTTGGATTTTG
GATTTTCAGAGGAGCATTGGCGTTAGGGTTTAAGATTTTCGTGGGAGCCCTACATGCACAGT
TGGCTTAGAAAGGGAGGTGCCCAACGACAACGATCGGTTTCGGCGGTGGTGGGAGTTGAGG
GACGGCAGGACGCACCGCCGAGCAGGCAGTGGAAGAACTATTGGGGGTGGGGGCAAGGCG
GCAACAAATTCGCCTCCGTCCTTCCATCTCTACCAAATTGCTCGTGCAATCACAACCACCTC
GTCGTTGCGCATCGCCTAGACGCACCCAAAAGGTCCTGCTTCGTCGGAGTTGACCGC

>Ea-0267

CCGTTGTCTGCGGCCGTAGGGGCAGGGGAAGGACCCGGCGCGGGAAGCGGAAGGATGGCGGA
AATAGGCCCACTATTCCTAATTTAGGTTCTATAGGGTG GGGCCCCGTCTCGCCTCCCGTTCGG

>Ea-0265

GAGCGCCTTGCCGAGGCTCGGCAGAGGATGGCTGCGCTTGGTGACGAGCCAGTATGTGCGCA
TAAGAAGTGATGCAGCATAAAAGAGTTGGAAGAATCACCAGCATTATTACAGCACAATCAGAG
TGAAATTCAAATTTCAACATGAGGCCTTTTTAGCAAGCCTCAGTGAAGAAAATACTTGATTCGG
GCACTGTTTACTCGCTTTCCCTTTGGGCTAGTTGTTCTAATTGATGGTTAACTAGTTGATGAAGAA
